# Adipose specific disruption of seipin causes early-onset generalised lipodystrophy and altered fuel utilisation without severe metabolic disease

**DOI:** 10.1016/j.molmet.2018.01.019

**Published:** 2018-01-31

**Authors:** George D. Mcilroy, Karla Suchacki, Anke J. Roelofs, Wulin Yang, Yanyun Fu, Bo Bai, Robert J. Wallace, Cosimo De Bari, William P. Cawthorn, Weiping Han, Mirela Delibegović, Justin J. Rochford

**Affiliations:** 1 The Rowett Institute, University of Aberdeen, Aberdeen, UK; 2 The Queen's Medical Research Institute, University of Edinburgh, Edinburgh, UK; 3 Institute of Medical Sciences, University of Aberdeen, UK; 4 Cancer Hospital and Anhui Province Key Laboratory of Medical Physics and Technology, Hefei Institutes of Physical Science, Chinese Academy of Sciences, Anhui, China; 5 Laboratory of Metabolic Medicine, Singapore Bioimaging Consortium, Agency for Science, Technology and Research (A*STAR), Singapore; 6 Department of Orthopaedics, University of Edinburgh, Edinburgh, UK

**Keywords:** BSCL2, Seipin, CGL2, Lipodystrophy, Adipose tissue, Browning

## Abstract

**Objective:**

Mutations to the *BSCL2* gene disrupt the protein seipin and cause the most severe form of congenital generalised lipodystrophy (CGL). Affected individuals exhibit a near complete loss of white adipose tissue (WAT) and suffer from metabolic disease. Seipin is critical for adipocyte development in culture and mice with germline disruption to *Bscl2* recapitulate the effects of *BSCL2* disruption in humans. Here we examined whether loss of *Bscl2* specifically in developing adipocytes *in vivo* is sufficient to prevent adipose tissue development and cause all features observed with congenital *BSCL2* disruption.

**Methods:**

We generated and characterised a novel mouse model of *Bscl2* deficiency in developing adipocytes (Ad-B2^(−/−)^) using the adipose-specific Adiponectin-Cre line.

**Results:**

We demonstrate that Ad-B2^(−/−)^ mice display early onset lipodystrophy, in common with congenital *Bscl2* null mice and CGL2 patients. However, glucose intolerance, insulin resistance, and severe hepatic steatosis are not apparent. Food intake and energy expenditure are unchanged, but Ad-B2^(−/−)^ mice exhibit significantly altered substrate utilisation. We also find differential effects of seipin loss between specific adipose depots revealing new insights regarding their varied characteristics. When fed a high-fat diet, Ad-B2^(−/−)^ mice entirely fail to expand adipose mass but remain glucose tolerant.

**Conclusions:**

Our findings demonstrate that disruption of *Bscl2* specifically in developing adipocytes is sufficient to cause the early-onset generalised lipodystrophy observed in patients with mutations in *BSCL2*. However, this significant reduction in adipose mass does not cause the overt metabolic dysfunction seen in *Bscl2* knockout mice, even following a high-fat diet challenge.

## Highlights

• Seipin loss only in developing adipocytes drives severe early-onset lipodystrophy.
• This leads to significantly altered use of metabolic substrates.
• We uncover developmental differences between poorly characterised adipose depots.
• Despite severely reduced adipose mass mice do not show overt metabolic disease.

## 1 Introduction

Congenital generalised lipodystrophy (CGL) is a rare, autosomal recessive, genetic disorder characterised by dramatically reduced adipose tissue mass from birth [1]. Individuals with CGL develop hepatic steatosis, hyperlipidaemia, and severe insulin resistance. This is predicted to result from the inability to increase the number of new adipocytes by adipogenesis and/or expand existing adipose cells to safely store dietary nutrients, principally as triglyceride. So far, mutations in four genes have been identified that cause CGL: *AGPAT2* (CGL type 1) [2], *BSCL2* (CGL type 2) [3], *CAV1* (CGL type 3) [4], and *PTRF* (CGL type 4) [5]. A mutation in the promoter of the *FOS* gene is also associated with a generalised lipodystrophy [6]. Disruptions to *AGPAT2* and *BSCL2* account for around 95% of all reported cases of CGL of known genetic cause. Patients with homozygous or compound heterozygous loss of function mutations in *BSCL2* exhibit the most severe form, CGL2 [7]. Affected individuals almost entirely lack both metabolic and mechanical adipose tissue depots and typically suffer from severe insulin resistance, dyslipidemia, hepatic steatosis, and hyperphagia due to extremely low circulating levels of the satiety hormone leptin [8]. *BSCL2* is highly expressed in adipose tissue; however, it is also expressed in other tissues with the highest expression levels being found in the testes and brain [3], [9]. *BSCL2* encodes the protein seipin, an endoplasmic reticulum (ER) transmembrane protein capable of forming homo-oligomers that may act as molecular scaffolds and/or directly modulate lipid droplet dynamics [10], [11]. Cellular studies have revealed that knockdown of *Bscl2* impairs adipogenesis and provided the first mechanistic insights to the cause of CGL2 [12], [13]. Seipin has been reported to interact with multiple proteins via which it may influence adipogenesis, including regulators of triacylglycerol/glycerophospholipid synthesis (LIPIN1, AGPAT2 and GPAT3) [14], [15], [16], ER calcium signalling (SERCA) [17] and cytoskeletal organisation [18]. Despite these advances, the exact molecular function of seipin remains uncertain. Three independent laboratories have generated mouse models with germline disruption to *Bscl*2 [19], [20], [21]. All models developed severe lipodystrophy, confirming the requirement of seipin for adipose tissue development and/or maintenance. *Bscl2* null mice exhibit insulin resistance and almost all other phenotypic characteristics of CGL2 patients, with the exception of hypertriglyceridemia [7], although this is also observed with *Bscl2* null mice also lacking the low density lipoprotein receptor [22]. Re-expression of seipin in adipocytes under the control of the aP2 promoter in *Bscl2* null mice rescues lipodystrophy, insulin resistance and hepatic steatosis [23]. However, this does not specifically demonstrate that the loss of seipin only in developing adipose tissue can drive the full CGL phenotype. Moreover, the analysis is complicated by the known capacity of the aP2 promoter to drive expression in non-adipose tissues [24], [25]. The aP2-Cre model has also been used to selectively ablate *Bscl2* in adipocytes but, unlike congenital seipin loss in mice and humans, this does not significantly alter adipose mass in early life and instead leads to a progressive loss of adipose tissue in adulthood [26]. A tamoxifen-inducible *Adipoq*-CreER model has been used to examine the effects of acute loss of seipin in mature adipocytes in adult mice, revealing valuable insights regarding the importance of seipin for adipocyte maintenance and function [27]. However, tamoxifen itself can influence adipose development and function and therefore can confound interpretation when used in such studies [28]. In this study, we have used the non-inducible *Adipoq*-Cre model to investigate the consequences of ablating *Bscl2* in developing and mature adipocytes. This strategy has been shown to efficiently target developing adipocytes in other *in vivo* studies of key adipogenic genes [29], [30]. Our data reveal that seipin deficiency in developing adipocytes is sufficient to cause severe generalised lipodystrophy early in life but that this does not induce all of the metabolic consequences of congenital seipin deficiency.

## 2 Methods

### 2.1 Animal studies

Bscl2^tm1a(EUCOMM)Hmgu^ mice were generated using ES cells sourced from EUCOMM [31]. The LacZ/Neo cassette preceding exon 5 was removed by Flp/Frt recombination to generate mice in which LoxP sites were inserted either side of exons 5–7 of *Bscl2* (*Bscl2*^(fl/fl)^ mice). To generate seipin knockout mice (SKO), Bscl2^tm1a(EUCOMM)Hmgu^ mice were crossed with oocyte-specific ZP3-Cre transgenic mice. Experimental SKO mice were generated by crossing heterozygous mice and analyses were performed with male SKO mice and male wild-type littermates. To generate an adipocyte-specific model of seipin deficiency (Ad-B2^(−/−)^), homozygous *Bscl2*^(fl/fl)^ mice were crossed with heterozygous *Bscl2*^(fl/wt)^ mice also containing Cre recombinase driven by the *Adipoq* promoter (Adiponectin-Cre). Adiponectin-Cre mice were generously provided by Dr Evan Rosen, Beth Israel Deaconess Medical Center, Harvard Medical School, Boston, USA. Animal procedures conducted on Ad-B2^(−/−)^ mice were approved by the University of Aberdeen Ethics Review Board and performed under a project license (PPL: P94B395EO) approved by the UK Home Office. All experiments used male mice, which were group-housed at 20–22 °C and exposed to a 12hr/12hr light–dark period. Male littermates heterozygous for both Adiponectin-Cre and *Bscl2*^(fl/wt)^ were used as controls (Ad-B2^(+/−)^). Mice were always given *ad libitum* access to water and a standard rodent chow diet (CRM (P) 801722, Special Diets Services) unless otherwise stated. Tissues were rapidly dissected post-mortem, frozen in liquid nitrogen then stored at −70 °C.

### 2.2 Metabolic studies

Fat and lean mass were measured in mice aged 6 and 12 weeks (chow diet) or 8, 10, and 12 weeks of age (high-fat diet) using the EchoMRI™-500 body composition analyser (Zinsser Analytic GmbH) then normalised to body weight. Food intake, water intake, activity, energy expenditure (EE), and respiratory exchange ratio (RER) were continuously measured using the PhenoMaster/LabMaster Home Cage System (TSE Systems). Mice were individually housed and acclimatised for one week prior to metabolic measurements being taken. Prior to glucose tolerance tests (GTT), mice were placed into clean cages, and food was withheld for 5 h. Basal glucose readings (0 min) were determined by glucometer readings (AlphaTrak^®^ II, Zoetisus) from tail punctures. Mice were then given a 2 mg/g d-glucose (Sigma) bolus by intraperitoneal injection. Blood glucose levels were monitored at 15, 30, 60, and 120 min. Mice had *ad libitum* access to water throughout. A separate cohort of eight-week-old Ad-B2^(−/−)^ and Ad-B2^(+/−)^ littermate controls were group-housed and placed on a high-fat diet (60% kcals from fat (D12492), Research Diets) for four weeks. All mice had *ad libitum* access to food and water unless otherwise stated.

### 2.3 Histology

Immediately following CO_2_ anaesthesia and cervical dislocation, epididymal white adipose tissue (EWAT), subcutaneous white adipose tissue (SWAT), and brown adipose tissue (BAT) were immersed in 10% formalin and stored at 4 °C for 24 h. Tissues were then transferred to PBS. Tissues were processed on a Peloris Tissue Processor (Leica) and were embedded into Sakura Tissue-Tek III paraffin wax (melting point 54–57 °C). Sections were cut at 4 μm intervals and dried at 37 °C overnight. Sections were then stained with H&E. Caudal vertebrae were fixed in formalin at 4 °C for one week, washed in PBS, and then decalcified in 14% EDTA for 14 days at 4 °C. Caudal vertebrae were paraffin wax-embedded, 6 μm sections were taken every 100 μm and dried at 37 °C overnight. Sections were stained with H&E, and the size distribution of marrow adipocyte area was determined by manual counting using Image J software, 300 adipocytes were counted per animal. Knees were fixed in 4% methanol-free paraformaldehyde (PFA; TAAB, Reading, UK) overnight, followed by decalcification in 4–10% EDTA (WVR Chemicals, Leuven, Belgium) for 2–4 weeks at 4 °C. They were then dehydrated, embedded in paraffin, and cut into 5 μm-thick sagittal sections. For each knee, sections were selected for analysis using the posterior cruciate ligament as central landmark. Sections were rehydrated, stained with H&E, and tile-scanned on a Zeiss Axioscan Z1 slide scanner (Carl Zeiss Ltd). Average IFP surface area and average number of adipocytes were determined manually from 2 to 3 H&E-stained sections per knee using ZEN2010 software (Carl Zeiss Ltd) by an experimenter blinded to the mouse genotype.

10 μm sections of frozen livers were cut at −20 °C, fixed for 10 min in formalin, rinsed in PBS, and then stained with oil red O essentially as described in [12].

### 2.4 Marrow adipose tissue quantification by osmium staining and μCT

Tibiae were isolated and, after removal of external soft tissue, fixed in 10% formalin for 2–4 days at 4 °C. Fixed tibiae were decalcified in 14% EDTA for 14 days and then washed in Sorensen's Phosphate buffer (81 mM KH2PO4, 19 mM Na2HPO4 ⋅ 7H2O, pH 7.4). Decalcified tibiae were stored in Sorensen's Phosphate buffer at 4 °C until ready to be stained with osmium tetroxide. To do so, osmium tetroxide solution (2% w/v; Agar Scientific, UK) was diluted 1:1 in Sorensen's Phosphate buffer. Tibiae were then stained in this 1% osmium tetroxide solution for 48 h at room temperature, washed, and stored in Sorensen's Phosphate buffer at 4 °C. For micro computed tomography (μCT) analysis, layers of four to five stained tibiae were arranged in parallel in 1% agarose in a 30-mL universal tube and mounted in a Skyscan 1172 desktop micro CT (Bruker, Kontich, Belgium). The samples were then scanned through 360° using a step of 0.40° between exposures. A voxel resolution of 12.05 μm was obtained in the scans using the following control settings: 54 kV source voltage, 185 μA source current with an exposure time of 885 ms. A 0.5-mm aluminium filter and two-frame averaging were used to optimize the scan. After scanning, the data were reconstructed using Skyscan software NRecon v1.6.9.4 (Bruker, Kontich, Belgium). The reconstruction thresholding window was optimized to encapsulate the target image. Volumetric analysis was performed using CT Analyser v1.13.5.1 (Bruker, Kontich, Belgium).

### 2.5 Gene expression

Total RNA was extracted from frozen tissues using the RNeasy mini kit (Qiagen) following the manufacturer's protocol, or a standard guanidinium thiocyanate-phenol-chloroform extraction protocol. Equal quantities of RNA were DNase I treated (Sigma) then reverse transcribed with M-MLV reverse transcriptase, 5X reaction buffer, dNTP's and random primers (Promega). Real-time quantitative PCR was performed on the 7900HT system (Applied Biosystems) or CFX384 Touch™ Real-Time PCR Detection System (BioRad). NTC and NoRT controls were performed for every gene analysed. The geometric mean of three stable reference genes (*Nono*, *Ywhaz*, and *Hprt*) was used for normalisation.

### 2.6 Western blotting

Frozen BAT tissues were homogenised in RIPA lysis buffer containing cOmplete™ protease inhibitor cocktail (Roche). Protein concentrations were determined by BCA assay (Thermo). SDS-PAGE was performed using equal quantities of protein which were transferred to PVDF membrane using standard protocols. Antibodies used included anti-Bscl2 (ab106793, Abcam) and anti-Calnexin (ab75801, Abcam). Anti-Rabbit HRP secondary antibody was used (Cell Signaling) and was visualized using enhanced chemiluminescence (ECL substrate, Illuminata) and quantified by densitometry scanning using Image J software.

### 2.7 Serum analysis

Blood was collected from 16-week old chow fed mice fasted for 5 h by cardiac puncture, placed and inverted in SST™ amber tubes (BD Microtainer^®^) and incubated at room temperature for 30 min. Samples were then centrifuged at 12,000×*g* for 10 min and the separated serum collected. Insulin, adiponectin, and leptin analyses were performed at the Core Biochemical Assay Laboratory (Cambridge, UK). Glucose levels were determined using the Glucose Coulometric Assay Kit (Cayman Chemical) and following the manufacturer's protocol provided. Serum triglyceride levels were determined using the Triglyceride Liquid Assay (Sentinel Diagnostics) following the manufacturer's instructions.

Quantitative insulin sensitivity check index (QUICKI) was calculated from fasting glucose (mg/dL) and insulin (μU/mL) values as previously described [32]. QUICKI = 1/[log(I_0_) + log(G_0_)], where I_0_ is fasting insulin and G_0_ is fasting glucose. QUICKI is a dimensionless index without units.

### 2.8 Liver TG assay

Frozen liver tissue samples were weighed and then homogenised in 1 ml of PBS. Samples were kept on ice at all times. Liver lysates were centrifuged at 12,000×*g* for 10 min at 4 °C. The supernatant was collected and triglyceride (TG) levels were determined using the Triglyceride Liquid Assay (Sentinel Diagnostics) following the manufacturer's instructions. TG levels were then normalised to individual tissue weights.

### 2.9 Statistical analyses

All data are presented as the mean ± SEM and were analysed by an unpaired two-tailed Student's *t* test or two-way analysis of variance with Bonferroni post-hoc test as appropriate using GraphPad Prism. Mendelian frequencies were analysed using a Chi-Square test. A *P*-value <0.05 was considered as statistically significant.

## 3 Results

### 3.1 Deletion of *Bscl2* specifically in developing adipocytes is sufficient to cause lipodystrophy

**Figure 1:**
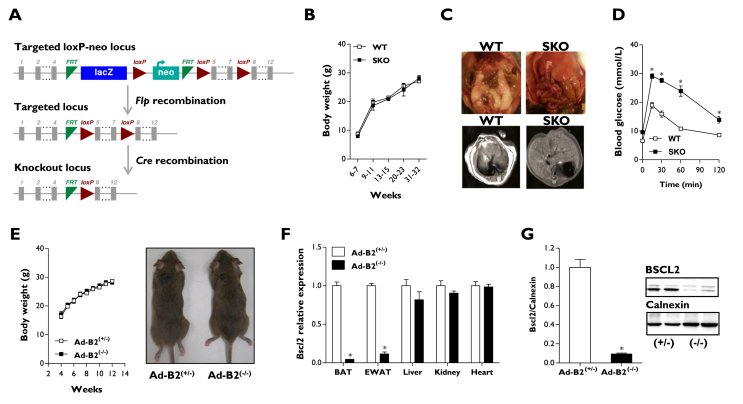
**Generation of Ad-B2**^**(−/−)**^**mice.** (A) Targeting strategy for the conditional disruption of the *Bscl2* gene. Body weights (B), dissection images with magnetic resonance imaging (C), and glucose tolerance (D) of SKO mice (n = 5–6). (E) Body weights and gross morphology of mice after Adiponectin-Cre recombination (n = 7–8). (F) *Bscl2* mRNA levels of 16-week-old Ad-B2^(+/−)^ and Ad-B2^(−/−)^ mice in BAT, EWAT, liver, kidney, and heart (n = 4–5). (G) Western blot analysis of Bscl2 protein from BAT of mice described in F (n = 4–5). All data are presented as the mean ± SEM, *p < 0.05.

**Figure 2:**
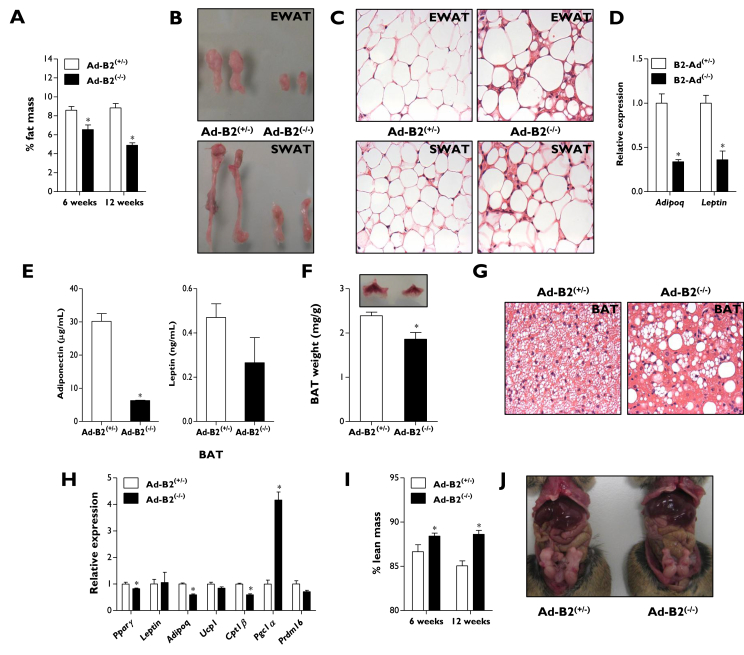
**Characterisation of Ad-B2**^**(−/−)**^**mice.** (A) Fat mass normalised to body weight of Ad-B2^(+/−)^ and Ad-B2^(−/−)^ mice at 6 (n = 4–7) and 12 weeks of age (n = 7–8). Dissection images (B) and representative 40× magnification images of H&E staining (C) of EWAT and SWAT sections of 12-week-old Ad-B2^(+/−)^ and Ad-B2^(−/−)^ mice. Relative EWAT mRNA levels (D) and circulating serum levels (E) of adiponectin and leptin in 16-week-old Ad-B2^(+/−)^ and Ad-B2^(−/−)^ mice (n = 4–5) fasted for 5 h. (F) Dissection image and BAT weight normalised to body weight (n = 4–5). (G) Representative 40× magnification images of H&E staining of BAT sections of 12-week-old Ad-B2^(+/−)^ and Ad-B2^(−/−)^ mice. (H) mRNA levels of white and brown markers in BAT of 16-week-old Ad-B2^(+/−)^ and Ad-B2^(−/−)^ mice (n = 4–5). (I) Lean mass at 6 (n = 4–7) and 12 weeks of age (n = 7–8) normalised to body weight, and gross abdominal morphology (J) of 16-week-old Ad-B2^(+/−)^ and Ad-B2^(−/−)^ mice. All data are presented as the mean ± SEM, *p < 0.05.

To investigate the consequences of disrupting *Bscl2*, we used a novel mouse model generated from Bscl2^tm1a(EUCOMM)Hmgu^ ES cells in which exons 5–7 of the *Bscl2* gene are flanked by LoxP sites (Figure 1A). To validate this model, we first generated global seipin knockout (SKO) mice. SKO mice did not display significant differences in body weight (Figure 1B) but developed lipodystrophy (Figure 1C), glucose intolerance (Figure 1D), and insulin insensitivity (Figure S1A), phenocopying previously reported seipin knockout models [19], [20], [21]. Subsequently, *Bscl2*-floxed mice were used to generate mice lacking seipin selectively in developing and mature adipocytes (Ad-B2^(−/−)^ mice) by crossing with the adipose specific Adiponectin-Cre model [33]. Ad-B2^(−/−)^ mice were born at the expected Mendelian frequencies (HE/WT 23.6%, HE/HE 25.7%, HO/WT 30.4% and HO/HE 20.3%, *n* = 237, *P* = 0.164). Similar to the SKO mice (Figure 1B), body weights and gross morphology of Ad-B2^(−/−)^ mice were not significantly different from littermate controls (Ad-B2^(+/−)^) when fed a standard chow diet (Figure 1E). The specificity of *Bscl2* deletion was confirmed by qPCR, which showed dramatically reduced mRNA levels in epididymal white adipose tissue (EWAT) and brown adipose tissue (BAT) but not in liver, kidney, heart (Figure 1F), or brain (data not shown). Western blot analysis furthermore indicated that seipin protein levels were significantly depleted in BAT of Ad-B2^(−/−)^ mice (Figure 1G). Consistent with a critical role for seipin in WAT development and/or maintenance *in vivo*, Echo-MRI analysis revealed knockout mice had significantly decreased fat mass levels at both six and twelve weeks of age (Figure 2A) and only residual EWAT and subcutaneous WAT (SWAT) remained (Figure 2B). H&E staining of sections from the residual EWAT and SWAT indicated that Ad-B2^(−/−)^ mice had a disorganised mixture of hypertrophic and smaller adipocytes (Figure 2C). Gene expression levels of *Adipoq* and *Leptin* were significantly reduced in EWAT of Ad-B2^(−/−)^ mice (Figure 2D) and serum analysis in fasted mice revealed significantly decreased circulating adiponectin levels (Figure 2E), although circulating leptin levels were not significantly reduced under these conditions (Figure 2E & S1B). Despite a greater than 90% decrease in *Bscl2* mRNA and seipin protein expression (Figure 1F–G), BAT in Ad-B2^(−/−)^ mice showed only a modest reduction in total mass (Figure 2F). However, H&E staining of sections from BAT revealed altered triglyceride accumulation in Ad-B2^(−/−)^ mice, with numerous large droplets rather than the normal multilocular morphology observed in BAT of littermate controls (Figure 2G). Modest, significant decreases in *Pparγ*, *Adipoq*, and *Cpt1β* gene expression were found in BAT, but no differences in *Leptin*, *Ucp1* or *Prdm16* were observed. Interestingly, a ∼4 fold increase was observed in *Pgc1α* expression (Figure 2H). Lean mass was significantly increased in Ad-B2^(−/−)^ mice at both 6 and 12 weeks of age (Figure 2I), a feature of congenital seipin loss in mice [20]. In contrast, adipose-specific loss of seipin did not lead to generalised organomegaly (Figure 2J), which is also typically observed in seipin knockout mice, and kidney or heart weights were not significantly different between genotypes (Figure S1C). The severe hepatic steatosis seen in SKO mice (Figure 1C) and CGL2 patients [34] was also not apparent in Ad-B2^(−/−)^ mice (Figure 2J). Overall, these findings reveal that loss of *Bscl2* specifically in developing adipocytes is sufficient to cause generalised lipodystrophy in early life *in vivo*, leading to several, but not all, clinical features observed in CGL2 patients and global *Bscl2* knockout mice.

### 3.2 Analysis of metabolic and mechanical adipose tissues in Ad-B2^(−/−)^ mice reveals depot-selective characteristics

CGL2 is notable for the loss of so-called “mechanical” adipose depots, which are selectively retained in other forms of CGL. Hence, we next determined the severity of adipose tissue loss due to *Bscl2* ablation in some less frequently examined metabolic and mechanical adipose depots.

**Figure 3:**
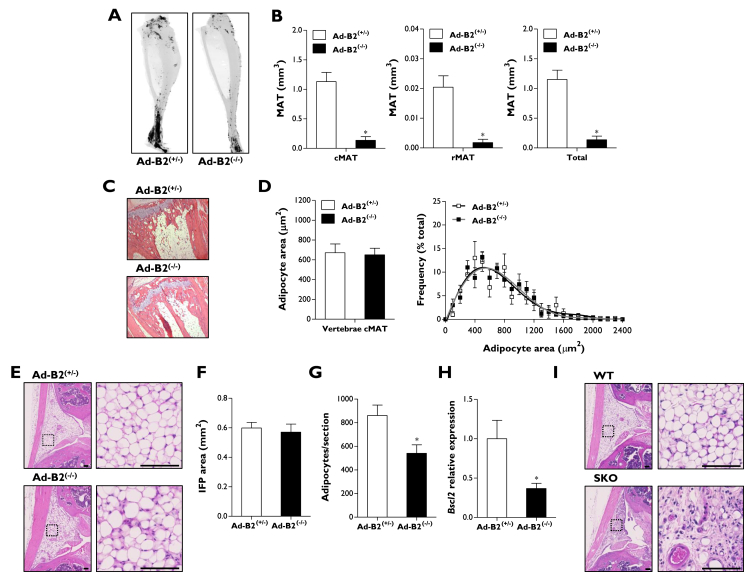
**Metabolic and mechanical adipose tissue in Ad-B2**^**(−/−)**^**mice.** (A) Whole mouse tibiae stained with osmium tetroxide and (B) quantification of cMAT, rMAT, and total MAT by μCT in 16-week-old Ad-B2^(+/−)^ and Ad-B2^(−/−)^ mice (n = 4–5). Representative H&E staining of MAT from caudal vertebrae from the tail (C) and quantification of adipocyte area and frequency of caudal vertebrae MAT (D) of mice described in A. (E) Representative H&E staining images of IFP sections in 12-week-old Ad-B2^(+/−)^ and Ad-B2^(−/−)^ mice. Scale bar represents 100 μm. Quantification of IFP size (F) and adipocytes per section (G) of mice described in E (n = 7). (H) *Bscl2* mRNA levels of 12-week-old Ad-B2^(+/−)^ and Ad-B2^(−/−)^ mice in IFP (n = 7). (I) Representative H&E staining images of IFP sections in WT and SKO mice. Scale bar represents 100 μm. All data are presented as the mean ± SEM, *p < 0.05.

We first examined bone marrow adipose tissue (MAT), which has been shown to be a highly metabolically active depot, capable of secreting large quantities of adiponectin [35]. Osmium tetroxide staining of whole tibiae from 16-week-old Ad-B2^(−/−)^ mice (Figure 3A) and quantification of MAT by μCT revealed severe depletion of total tibial MAT in Ad-B2^(−/−)^ mice, resulting from loss of both constitutive MAT (cMAT) of the distal tibia and regulated MAT (rMAT) in the mid-to-proximal tibia (Figure 3B). We next examined the MAT located in the caudal vertebrae of the tail, which has also been considered a region of cMAT [36]. Surprisingly, adipocytes were readily apparent in this region (Figure 3C) and were not significantly different in terms of the adipocyte area or adipocyte size distribution (Figure 3D). This suggests that vertebral cMAT may be developmentally and/or functionally distinct to cMAT in tibiae and other long bones.

To examine a depot classically thought of as “mechanical” adipose tissue, we analysed the infrapatellar fat pad (IFP) from the knee joint (Figure 3E). Although the size of the IFP was found to be not different between genotypes (Figure 3F), there was a modest but significant reduction in total number of IFP adipocytes in Ad-B2^(−/−)^ mice indicating a subtle disruption in this depot is apparent in this model (Figure 3G). However, the IFP was largely spared, which was unexpected given the observed loss of mechanical adipose depots in CGL2 patients [34]. We examined whether the retention of IFP adipocytes in Ad-B2^(−/−)^ mice could result from less efficient targeting of this depot by the Adiponectin-Cre model. Consistent with this, the expression of *Bscl2* mRNA was reduced by ∼60% in the IFP of Ad-B2^(−/−)^ mice (Figure 3H) compared with the 90% or greater reduction observed in other adipose depots examined (Figure 1F). To determine whether seipin was indeed needed for IFP development, we examined the IFP in our SKO mice. This revealed that adipocytes of the IFP were almost completely absent in these global seipin knockout mice (Figure 3I). Taken together, our findings reveal that adipose-specific ablation of *Bscl2* using the Adiponectin-Cre line causes early-onset generalised loss of adipose tissue but that a small number of specific depots, such as the IFP, may be poorly targeted by the Adiponectin-Cre model.

### 3.3 Severe metabolic dysfunction is not apparent in Ad-B2^(−/−)^ mice

**Figure 4:**
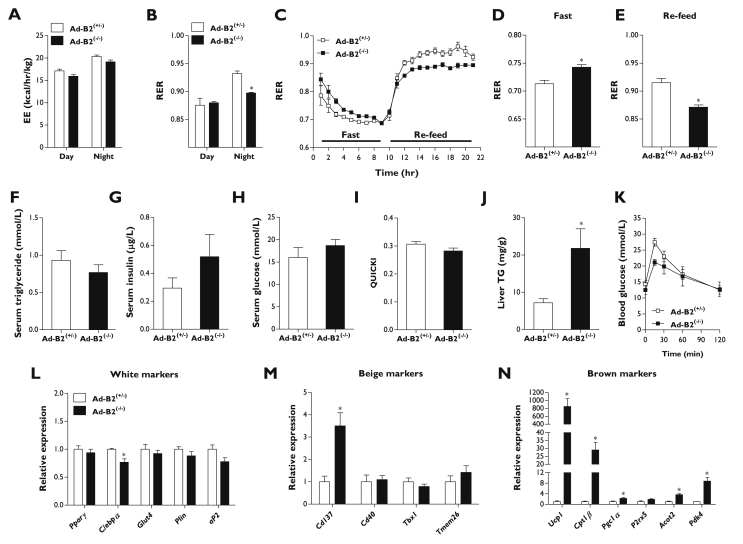
**Physiological and metabolic phenotype of Ad-B2**^**(−/−)**^**mice.** (A) Energy expenditure (EE) and (B) respiratory exchange ratio (RER) values in Ad-B2^(+/−)^ and Ad-B2^(−/−)^ mice at 14 weeks of age fed a standard chow diet (n = 4–5). (C) Mice as described in B were challenged with a 9-h fast and then given *ad libitum* access to chow diet and RER values were collected throughout. Average RER values obtained during fasting (D) and re-feeding (E) periods (n = 4–5). Analysis of serum triglyceride (F), insulin (G), glucose (H), quantitative insulin sensitivity check index (QUICKI) analysis (I) and liver triglyceride levels (J) normalised to tissue weight in Ad-B2^(+/−)^ and Ad-B2^(−/−)^ mice fasted for 5 h at 16 weeks of age (n = 4–5). (K) Glucose tolerance test in Ad-B2^(+/−)^ and Ad-B2^(−/−)^ mice at 10 weeks of age (n = 4). Relative gene expression of white (L), brite (M) and brown (N) adipocyte markers in EWAT of Ad-B2^(+/−)^ and Ad-B2^(−/−)^ mice at 16 weeks of age (n = 4–5). All data are presented as the mean ± SEM, *p < 0.05.

To examine the metabolic consequences of adipose-specific ablation of seipin, 14-week-old Ad-B2^(−/−)^ mice were phenotyped in metabolic cages. Food and water intake was not significantly different from littermate controls (Figure S1D). Activity levels during the night were also not altered; however, Ad-B2^(−/−)^ mice were found to be significantly less active during the day (Figure S1E). Energy expenditure levels were not significantly altered between genotypes during either day or night (Figure 4A); however, knockout mice had significantly decreased respiratory exchange ratio (RER) values during the active phase (Figure 4B), indicating an increased propensity towards lipid oxidation. To further explore this, mice were challenged with a fast/re-feed experiment and RER levels were continuously monitored. During a 9-h fast, Ad-B2^(−/−)^ mice had elevated starting RER values compared to littermate controls (Figure 4C) and their mean RER levels remained higher during much of the fasting period (Figure 4D). Upon re-feeding, RER levels in both genotypes increased (Figure 4C), however, Ad-B2^(−/−)^ mice failed to increase their RER to the same level as control mice, leading to significantly lower mean values (Figure 4E). This is consistent with the data obtained during the active phase in *ad libitum* fed mice (Figure 4B). Overall, these data indicate that Ad-B2^(−/−)^ mice may have a reduced flexibility to switch between the use of lipids as fuel during fasting and carbohydrate upon refeeding. To determine if this was detrimental to metabolic health, we examined serum triglyceride, insulin, and glucose levels in 16-week-old mice that had been fasted for a period of 5 h. No significant differences were observed between control and Ad-B2^(−/−)^ mice (Figure 4F–H) and quantitative insulin sensitivity check index (QUICKI) analysis indicated that knockout mice were not insulin resistant compared to littermate controls (Figure 4I). Ad-B2^(−/−)^ mice did have elevated liver triglyceride levels (Figure 4J and S1F), most likely due to the absence of appropriate adipose tissue depots driving enhanced hepatic lipid accumulation. However, the increase in liver TG levels in Ad-B2^(−/−)^ mice was much less severe than that seen in previously reported Bscl2-null mice, consistent with the absence of overt liver steatosis in our model (Figure 2J). Glucose tolerance tests (GTTs) were performed to examine whether lipodystrophy and increased liver triglycerides would cause glucose intolerance. At the start of the GTT, 5-h fasted glucose levels were not significantly altered and, surprisingly, Ad-B2^(−/−)^ mice showed no impairment of glucose tolerance (Figure 4K).

SKO mice have been reported to show induction of markers of thermogenesis in residual EWAT deposits [20] and we next sought to determine if this feature was shared in Ad-B2^(−/−)^ mice. Although a small but significant decrease in *C/ebpα* was apparent, no changes were observed in the expression of other classical markers of WAT including, *Pparγ*, *Glut4*, *Perilipin* or *aP2* (Figure 4L). We next examined if the expression of markers characteristic of brite and brown adipocytes [37] were altered. When brite adipocyte markers (*Cd137*, *Cd40*, Tbx1, and *Tmem26*) were examined in Ad-B2^(−/−)^ mice, only *Cd137* was significantly elevated (Figure 4M). In contrast, expression of almost all markers of brown adipocytes that were examined were significantly increased, including *Ucp1*, *Cpt1β*, *Pgc1α*, *Acot2*, and *Pdk4*, suggesting browning occurs the residual EWAT depot in Ad-B2^(−/−)^ mice (Figure 4N).

Overall, our data indicate that the loss of *Bscl2* specifically in developing adipocytes causes lipodystrophy and ectopic lipid accumulation in the liver, along with significantly altered substrate utilisation in response to fasting and refeeding. However, this is not accompanied by the overt metabolic dysfunction seen in global *Bscl2* knockout mice.

### 3.4 High-fat-diet fed Ad-B2^(−/−)^ mice resist weight gain and remain glucose tolerant

**Figure 5:**
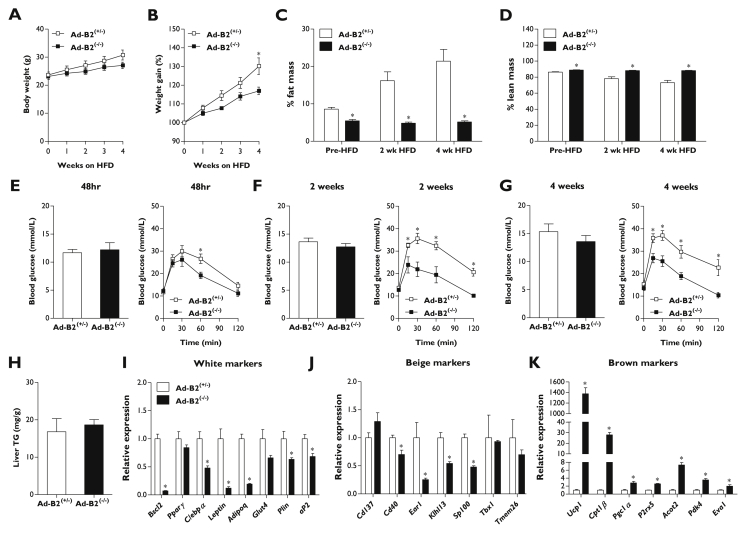
**Consequences of feeding Ad-B2**^**(−/−)**^**mice a high-fat diet.** Body weight (A) and percentage body weight gain (B) in Ad-B2^(+/−)^ and Ad-B2^(−/−)^ mice fed a high-fat diet (HFD) for four weeks, starting at 8 weeks of age (n = 5–6). Fat mass (C) and lean mass (D) normalised to body weight at 0, 2 and 4 weeks of HFD feeding (n = 5–6). Fasting glucose levels and glucose tolerance tests performed after 48 h (E), 2 weeks (F), and 4 weeks (G) of HFD feeding (n = 5–6). (H) Liver triglyceride levels normalised to tissue weight following 4 weeks of HFD feeding (n = 5–6). Relative gene expression of white (I), brite (J) and brown (K) adipocyte markers in EWAT of mice fed HFD for 4 weeks (n = 5–6). All data are presented as the mean ± SEM, *p < 0.05.

In an effort to uncover any latent metabolic dysfunction, 8-week-old Ad-B2^(−/−)^ mice and littermate controls were challenged with a high-fat diet (HFD, 60% kcal from fat) for four weeks. Ad-B2^(−/−)^ mice failed to gain as much weight as controls (Figure 5A). This was significantly lower compared to control mice after 4 weeks on HFD, when expressed as percentage of starting weight (Figure 5B). Echo-MRI analysis again revealed Ad-B2^(−/−)^ mice had significantly less fat mass than littermate control mice prior to HFD feeding. Strikingly, whilst adiposity more than doubled in control mice, Ad-B2^(−/−)^ mice were entirely unable to increase fat mass in response to HFD feeding (Figure 5C). Lean mass, as a percentage of body mass, was significantly increased in Ad-B2^(−/−)^ mice prior to HFD and remained elevated during HFD feeding. In contrast, the percentage lean mass in control mice steadily decreased when challenged with HFD as fat mass increased (Figure 5D). To determine if the inability to store triglycerides in adipose tissue was detrimental to metabolic health, intraperitoneal GTTs were performed at various times during the HFD challenge. In order to examine the acute effects of HFD, glucose tolerance was first examined after 48 h of HFD feeding. Five-hour fasted blood glucose levels were found to not be significantly altered and disposal rates of the glucose bolus were not different except at 60 min, when glycaemia in Ad-B2^(−/−)^ mice was significantly lower compared to control mice (Figure 3E). After two weeks of HFD feeding, basal fasted glucose levels were again not significantly different; however, Ad-B2^(−/−)^ mice exhibited improved glycaemia at all time-points examined compared to control littermates (Figure 5F). A final GTT was performed after four weeks of HFD to determine if this effect persisted. Once again, fasting blood glucose levels were found to be unaltered, while Ad-B2^(−/−)^ mice displayed significantly lower blood glucose levels after glucose bolus administration compared to littermate controls (Figure 5G). Ad-B2^(−/−)^ mice have a greater percentage lean mass and in these experiments glucose dosing was based on body weight. It is possible that the difference in body composition may bias the GTT to suggest greater glucose tolerance in the Ad-B2^(−/−)^ mice than is the case. However, previous studies of SKO mice have revealed marked glucose intolerance, despite also dosing GTT glucose based on total body weight [19], [21]. Hence, whilst one should be cautious about concluding that Ad-B2^(−/−)^ mice are more glucose tolerant than control mice, they are not glucose intolerant, like SKO mice.

When liver triglyceride levels were determined at the end of the HFD period, no significant differences were observed between the genotypes (Figure 5H). Comparison with absolute values for TG in chow fed mice (Figure 4J) indicated that this resulted from increased liver TG accumulation in control mice with HFD-feeding, whilst the elevated liver TG levels in chow-fed Ad-B2^(−/−)^ mice were not further increased substantially by HFD. Lipid accumulation, visualised by oil red O staining of liver sections from high-fat diet fed control and Ad-B2^(−/−)^ mice was consistent with assayed TG levels (Figure S1F).

Gene expression analysis of EWAT revealed either no change or significantly decreased transcript levels of both white and brite-specific markers in Ad-B2^(−/−)^ mice compared to controls (Figure 5I–J). Conversely, numerous markers of browning (*Ucp1*, *Cpt1β*, *Pgc1α*, *P2rx5, Acot2*, *Pdk4*, and *Eva1*) were all significantly elevated in Ad-B2^(−/−)^ mice (Figure 5K). Analysis of BAT revealed significant decreases in *Pparγ*, *Leptin*, *Adipoq, Ucp1, Cpt1β*, and *Prdm16* expression implying that the failure of HFD to worsen the metabolic health of Ad-B2^(−/−)^ mice was unlikely to result from increased lipid oxidation in BAT (Figure S1G). Overall, these results reveal that Ad-B2^(−/−)^ mice are unable to expand adipose tissue stores in response to a high-fat-diet challenge. Despite this, the detrimental consequences commonly associated with lipodystrophy in CGL2 are not observed.

## 4 Discussion

The human lipodystrophy gene *BSCL2* is known to be expressed in numerous tissues throughout the body [3]. Investigating the consequences of tissue-specific loss of seipin is therefore important to further understand and characterise this condition. We find that loss of seipin selectively in developing adipocytes in Ad-B2^(−/−)^ mice leads to several features seen in previously generated global *Bscl2* knockout mouse models including, critically, a severe lack of adipose tissue from early in life [19], [20], [21]. Consistent with this, circulating levels of the adipokine adiponectin were reduced. Moreover, Ad-B2^(−/−)^ mice challenged with a high-fat diet showed a striking failure to increase their adipose mass, revealing an inability to increase triglyceride storage or expand adipocyte number within their residual adipose tissues. These findings provide the first *in vivo* confirmation that loss of seipin specifically in developing adipocytes is sufficient to cause early-onset generalised lipodystrophy. In addition, our data show that seipin is important for adipose tissue expansion in adult mice, complementing a previous study of inducible ablation of seipin in adipose tissue in adults [27]. We observed significant alterations in fuel utilisation in Ad-B2^(−/−)^ mice as RER was higher than in controls during a period of fasting but did not rise to the same extent as in controls during re-feeding. We interpret this as an adaptation to an altered availability of stored lipids during fasting due to the paucity of adipose tissue, which may lead to a greater use of carbohydrate whilst glucose remains available. Conversely, if dietary lipids cannot be appropriately stored, they may make a greater contribution to fuel utilisation during re-feeding in Ad-B2^(−/−)^ mice, when control mice would instead incorporate more lipids into adipose tissue and make greater use of available carbohydrate. Future studies defining the mechanisms underlying this effect may provide novel insights regarding the switching between lipid and carbohydrate metabolism.

The phenotype we observe in Ad-B2^(−/−)^ mice, using the Adiponectin-Cre line, contrasts with a previous report using the aP2-Cre line to knock out seipin, which concluded that seipin loss in adipocytes resulted in a progressive lipodystrophy with apparently normal adipose tissue development in early life [26]. This is likely to result from the choice of Cre promoter used to ablate *Bscl2* from adipocytes. Indeed, our findings correlate with studies of adipose-selective ablation of PPARγ and the insulin receptor in which the use of Adiponectin-Cre model has revealed an almost complete loss of adipose tissue *in vivo* that is not evident using the aP2-Cre line [29], [30], [38], [39]. Overall, our work supports the findings of others that the Adiponectin-Cre line is preferable for examining the *in vivo* effects of targeting genes affecting adipose tissue development and function [24], [25].

Intriguingly, we find that whilst the Adiponectin-Cre line clearly targets all major white adipose depots, there may be some variation in targeting efficiency between some of the less studied depots. Bone marrow adipose tissue (MAT) is capable of secreting large quantities of adiponectin [35]. Consistent with this, *Bscl2* targeting in MAT of Ad-B2^(−/−)^ mice was highly efficient and led to severe depletion of both cMAT and rMAT stores in the tibia. Ad-B2^(−/−)^ mice therefore accurately model CGL2 in this regard [34] and may be particularly valuable for examining the consequences of severe MAT deficiency. However, it is notable that cMAT in the tail vertebrae was intact in the Ad-B2^(−/−)^ mice. Further studies are required to determine either whether these remaining adipocytes do not express adiponectin or if they do not require seipin for their development or maintenance. Regardless of the explanation for this, the selective maintenance of cMAT in the tail vertebrae implies that there is a fundamental difference in the development of axial vs appendicular MAT.

In contrast to tibial MAT but similar to vertebral MAT, we found that the IFP in the knee joint was largely preserved in Ad-B2^(−/−)^ mice. This depot is almost completely ablated in global seipin knockout mice (Figure 3H) and in CGL2 patients [40]. This strongly implies that the Adiponectin-Cre poorly targets this depot, at least early in adipogenesis, rather than that these adipocytes are unaffected by the absence of seipin. Consistent with this, we observed a more modest reduction in *Bscl2* expression in the IFP, in contrast to other adipose tissues in Ad-B2^(−/−)^ mice which showed a near-complete ablation of *Bscl2* expression. This adds to existing evidence that so-called “mechanical” WAT depots may differ from other depots, including the observation that this type of WAT is almost entirely absent in CGL2 patients but preserved in other forms of CGL [34], [40]. This type of WAT remains poorly understood but has been strongly implicated in joint disease [41]. Our data suggest that targeting strategies other than the Adiponectin-Cre model will be required to uncover its specific functions *in vivo*.

In addition to being lipodystrophic, Ad-B2^(−/−)^ mice displayed significantly increased lean mass, a feature also seen in *Bscl2* knockout mice [20]. However, in contrast to global KO mice, Ad-B2^(−/−)^ mice did not exhibit generalised organomegaly, and kidney or heart weights were not significantly altered. Hypertrophic cardiomyopathy and cardiac dysfunction in *Bscl2* knockout mice appears to be due to glucotoxicity [42]. The lack of an increase in heart mass in Ad-B2^(−/−)^ mice is consistent with this notion, as these mice are not hyperglycaemic or hyperinsulinaemic. How adipose-specific seipin deficiency and/or lack of adipose tissue drives increased lean mass remains an interesting unanswered question.

We were surprised to find that, despite generalised lipodystrophy, Ad-B2^(−/−)^ mice did not develop glucose intolerance or insulin resistance, even when mice were challenged with a high-fat diet. This finding is unexpected, as lipodystrophy resulting from adipose-tissue-specific ablation of PPARγ (PPARγ-FKO) or the insulin receptor (IR-FKO) using the Adiponectin-Cre line both result in severe metabolic dysfunction [29], [30]. Due to the critical role of PPARγ in brown adipogenesis, PPARγ-FKO mice lack BAT, whist in IR-FKO mice, BAT and any residual WAT will be insensitive to insulin. Previous studies have reported that *Bscl2* is not required for brown adipocyte differentiation or development [43], [44]. Consistent with this, Ad-B2^(−/−)^ mice developed interscapular BAT, albeit with modestly reduced mass, altered gene expression and lipid droplet morphology. Moreover, seipin-deficient brown adipocytes are not inherently insulin resistant, although they may become so indirectly in global seipin knockout mice [44]. Hence, preservation of BAT, and insulin sensitivity in BAT and residual WAT, may at least partly explain why Ad-B2^(−/−)^ mice do not develop the overt metabolic disease seen in PPARγ-FKO and IR-FKO mice.

In contrast, preserved BAT function and insulin sensitivity are unlikely to explain why Ad-B2^(−/−)^ mice are glucose tolerant whilst global *Bscl2* knockout mice are not. Both BAT development and browning of residual EWAT are also evident in *Bscl2* knockout mice but do not prevent overt metabolic disease, suggesting that other mechanisms must underlie this difference [19], [20], [21]. Also, the absolute mass of these residual EWAT depots is very small. Nonetheless, the browning of residual EWAT in all these seipin-deficient models is intriguing and might arise from the presence of bipotential precursor cells with the capacity to form BAT or WAT, which are forced by seipin deficiency towards brown adipogenesis. Given the array of distinct stem cells with adipogenic potential that have been reported, future lineage tracing studies may offer valuable insights regarding the nature of the adipocytes in these residual EWAT depots [45].

The restoration of *Bscl2* expression selectively in adipocytes of *Bscl2* knockout mice not only prevents lipodystrophy, but also insulin resistance and hepatic steatosis [23]. Although this indicates that these features of CGL2 are a consequence of the absence of appropriate adipose lipid storage capacity, they do not specifically address whether loss of seipin in non-adipose tissues may worsen metabolic health when adipose tissue is dysfunctional or absent. Our data raise the possibility that adipose tissue-specific ablation of *Bscl2* alone may be insufficient to cause all features of the severe metabolic disease observed in congenital seipin deficiency, at least in mice. One notable finding is that Ad-B2^(−/−)^ mice fail to develop severe hepatomegaly. Although liver triglycerides were elevated in chow-fed Ad-B2^(−/−)^ mice, overt hepatic steatosis was not observed and this was not exacerbated with high-fat diet feeding. *Bscl2* ablation specifically in the liver does not appear to lead to hepatic steatosis or diabetes, even when the mice are challenged with a high-fat diet [46]. However, adipose tissue mass was normal in these mice and may protect against the loss of *Bscl2* in the liver by safely storing additional fat and preventing ectopic hepatic triglyceride accumulation. A recent study has shown that seipin could play an important role in lipid storage within hepatocytes and that decreasing seipin expression in either primary or cultured hepatocytes increased the number and size of lipid droplets in these cells [47]. This appeared to be driven by greater fatty acid uptake and *de novo* lipogenesis. If seipin does have a cell-autonomous role regulating lipid handling within hepatocytes, this could explain the discrepancy in hepatic steatosis between Ad-B2^(−/−)^ and global *Bscl2* knockout mice. Hence, it remains plausible that seipin loss in hepatocytes, when adipose stores are also absent, could alter lipid homoeostasis and the regulation of gluconeogenesis, contributing to hyperglycaemia.

Nonetheless, it is also important to note that residual adipose tissue mass is greater in Ad-B2^(−/−)^ mice than in SKO mice, even if these remain quantitatively very small. This may still provide enough adipose storage capacity or adipokine secretion to protect the Ad-B2^(−/−)^ mice from metabolic disease. Further studies of ablation of seipin in non-adipose tissues of Ad-B2^(−/−)^ mice or additional ablation of mechanical adipose depots in these mice will be needed to determine whether this is the case.

In conclusion, we have generated a novel mouse model of adipose-specific *Bscl2* disruption. This reveals that the loss of seipin in developing adipocytes is sufficient to cause the early-onset generalised lipodystrophy observed in CGL2 patients with mutations in *BSCL2*. Moreover, substrate utilisation is significantly altered, demonstrating that the lack of adipose tissue drives metabolic adaptation in these mice. However, we do not observe the full phenotype of metabolic disease, particularly the severe hepatic steatosis, glucose intolerance and insulin resistance observed with congenital seipin deficiency in both humans and mice. This implies that there could be additional metabolically relevant roles for seipin in other tissues, and/or that recovery of quantitatively very modest adipose depots may be sufficient to alleviate metabolic disease in this rare but devastating condition.

## Author contributions

G.D.M. designed, performed and analysed experiments and wrote the manuscript. K.S., W.P.C. and A.J.R. designed, performed and analysed experiments. W.Y., Y.F., B.B. and R.J.W performed and analysed experiments. C.D.B., W.H and M.D. designed and interpreted experiments. J.J.R. conceived the study, designed and interpreted experiments, and wrote the manuscript. All authors reviewed the manuscript.

## Appendix A Supplementary data

Figure S1 **Supplementary data.** (A) Insulin tolerance test of SKO mice (n = 6). (B) Serum leptin in 16-week-old Ad-B2^(+/−)^ and Ad-B2^(−/−)^ mice (n = 4–5) fasted for 5 h adjusted to adipose mass in each mouse. (C) Kidney and heart weights normalised to body weight of 16 week old Ad-B2^(+/−)^ and Ad-B2^(−/−)^ mice (n = 4–5). Food intake, water intake (D) and activity levels (E) of Ad-B2^(+/−)^ and Ad-B2^(−/−)^ mice at 14 weeks of age fed a standard chow diet (n = 4–5). (F) Oil red O staining of sections of livers from 12 week old Ad-B2^(+/−)^ and Ad-B2^(−/−)^ mice fed a chow diet, or fed a high-fat diet (HFD) for four weeks. (G) mRNA levels of white and brown markers in BAT of Ad-B2^(+/−)^ and Ad-B2^(−/−)^ mice fed a high-fat diet (HFD) for four weeks (n = 5–6). All data are presented as the mean ± SEM, *p < 0.05.
