# Supplementary figures and images for: Adipose specific disruption of seipin causes early-onset generalised lipodystrophy and altered fuel utilisation without severe metabolic disease
Source: Mol Metab. 2018 Jan 31;10:55–65. doi: 10.1016/j.molmet.2018.01.019 (PMC5985228; doi:10.1016/j.molmet.2018.01.019)

## Slide 1
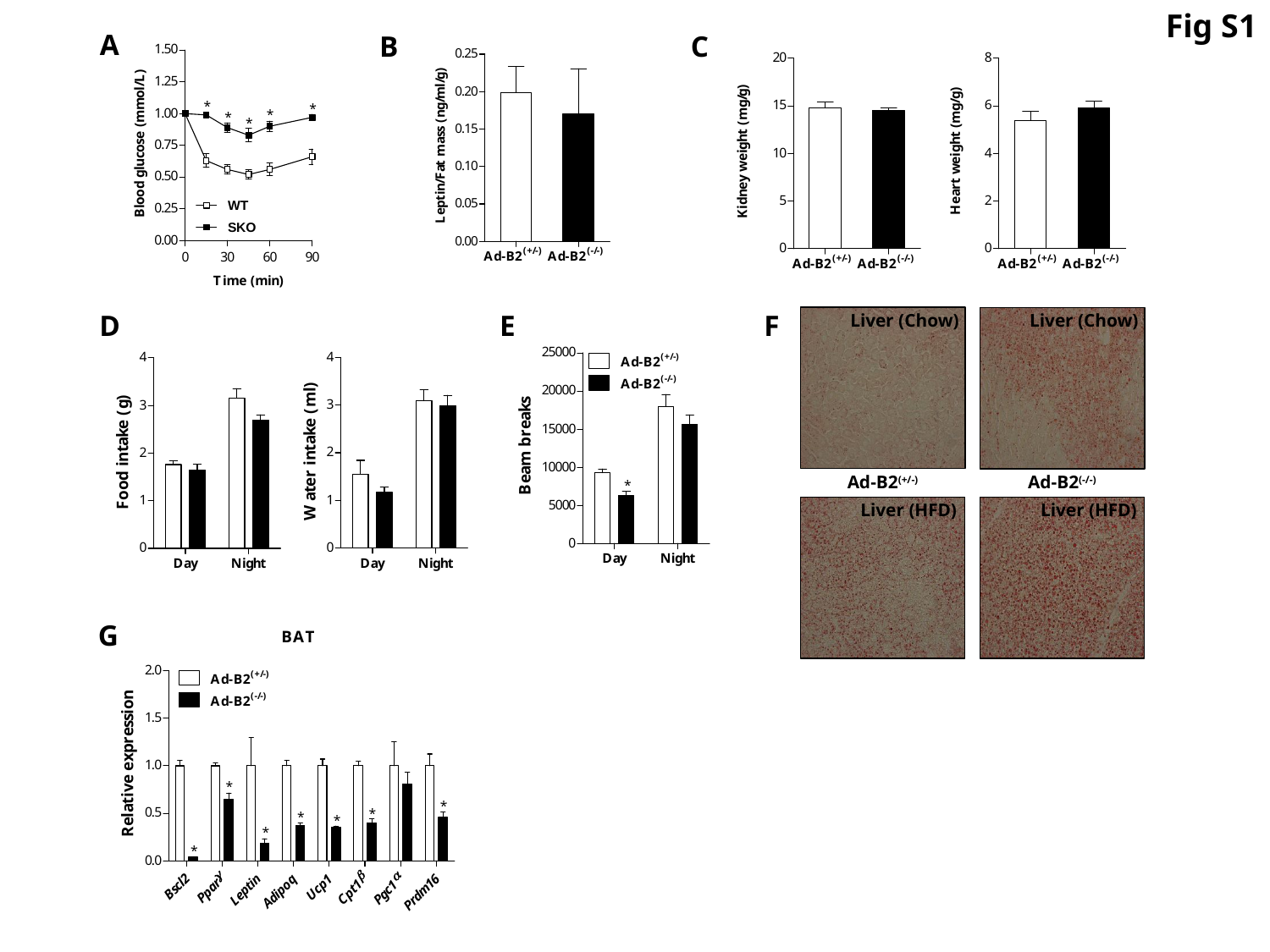

Fig S1
A
B
C
D
E
F
Liver (Chow)
Liver (Chow)
Ad-B2(+/-)
Ad-B2(-/-)
Liver (HFD)
Liver (HFD)
G

Supplement: Figure S1 — Supplementary data. (A) Insulin tolerance test of SKO mice (n = 6). (B) Serum leptin in 16-week-old Ad-B2(+/−) and Ad-B2(−/−) mice (n = 4–5) fasted for 5 h adjusted to adipose mass in each mouse. (C) Kidney and heart weights normalised to body weight of 16 week old Ad-B2(+/−) and Ad-B2(−/−) mice (n = 4–5). Food intake, water intake (D) and activity levels (E) of Ad-B2(+/−) and Ad-B2(−/−) mice at 14 weeks of age fed a standard chow diet (n = 4–5). (F) Oil red O staining of sections of livers from 12 week old Ad-B2(+/−) and Ad-B2(−/−) mice fed a chow diet, or fed a high-fat diet (HFD) for four weeks. (G) mRNA levels of white and brown markers in BAT of Ad-B2(+/−) and Ad-B2(−/−) mice fed a high-fat diet (HFD) for four weeks (n = 5–6). All data are presented as the mean ± SEM, *p < 0.05. [file mmc1.pptx]
